# Spontaneous Anterior Forearm Compartment Syndrome in a Healthy Postpartum Woman: A Rare Case

**DOI:** 10.7759/cureus.100933

**Published:** 2026-01-06

**Authors:** Aisha AlFarsi, Muzna AlSawafi

**Affiliations:** 1 Emergency Department, Medical City of Military and Security Services, Muscat, OMN

**Keywords:** fasciotomy, non-traumatic compartment syndrome, postpartum mothers, spontaneous presentation, sudden onset

## Abstract

Acute compartment syndrome typically follows trauma, fractures, or vascular compromise, with spontaneous presentations being exceedingly rare. We report a unique case of a 37-year-old healthy Middle Eastern woman, two months postpartum, who presented with sudden swelling and pain of the right forearm without any history of trauma or systemic disease. In this patient, several postpartum-specific physiological factors may have contributed to the underlying pathophysiology. Physical examination revealed significant volar forearm swelling and ecchymosis with preserved distal pulses. Imaging studies confirmed soft tissue swelling but excluded vascular occlusion or fracture, leading to a clinical diagnosis of anterior compartment syndrome. The patient underwent an urgent fasciotomy followed by three subsequent re-explorations for wound debridement. Her postoperative recovery was favorable, and she was discharged with arrangements for outpatient rehabilitation. This case underscores the rare occurrence of spontaneous compartment syndrome in the postpartum period. It highlights the critical importance of maintaining a high index of suspicion even in the absence of classic risk factors, as early recognition and surgical intervention are paramount to preventing long-term disability.

## Introduction

Acute compartment syndrome (ACS) is a surgical emergency characterized by increased pressure within a closed osteofascial compartment, leading to reduced tissue perfusion and potential irreversible muscle and nerve damage if not promptly treated [1]. Spontaneous cases without identifiable triggers are exceptionally rare. Such presentations have been documented in patients with underlying metabolic myopathies, such as McArdle disease [2], and in isolated reports of atraumatic forearm compartment syndrome [3]. Furthermore, ACS has been sporadically described following childbirth, though these instances almost exclusively involve the lower limbs [4]. In this context, several postpartum-specific physiological factors could potentially contribute to the development of spontaneous ACS. To our knowledge, spontaneous ACS occurring in the anterior forearm of a healthy postpartum woman without any discernible systemic risk factors has not been previously described in the literature.

## Case presentation

A 37-year-old healthy Middle Eastern woman, two months postpartum following an uncomplicated delivery, presented with acute right volar forearm swelling and severe pain that began spontaneously after she woke from a nap. She explicitly denied any history of trauma, strenuous activity, systemic illness, or use of anticoagulant medications.

On presentation, her vital signs were notable for a blood pressure of 152/69 mmHg, a heart rate of 86 beats per minute, and an oxygen saturation of 99% on room air, and she was afebrile. Physical examination of the right forearm revealed significant swelling with overlying ecchymosis along the distal volar aspect. Despite the swelling, distal radial and ulnar pulses were intact, and capillary refill was normal. A tentative passive stretch test elicited significant pain, raising the initial concern for compartment syndrome.

The initial laboratory investigation revealed a hemoglobin level of 10 g/dL, a white blood cell count of 8.22 × 10⁹/L, a C-reactive protein of 18.7 mg/L, a serum creatinine of 55 µmol/L, and a D-dimer level of 0.36 µg/mL. Radiographic imaging of the forearm showed soft-tissue swelling but no evidence of fracture. A Doppler ultrasound was performed, which demonstrated patent major vessels with diffuse cellulitic changes. Subsequently, a CT angiography confirmed preserved arterial flow throughout the forearm and hand, with no evidence of fractures or space-occupying masses. The initial evaluation at a regional hospital strongly suspected compartment syndrome, prompting an urgent referral to our tertiary center for secondary assessment and definitive management.

Management and surgical findings

**Figure 1:**
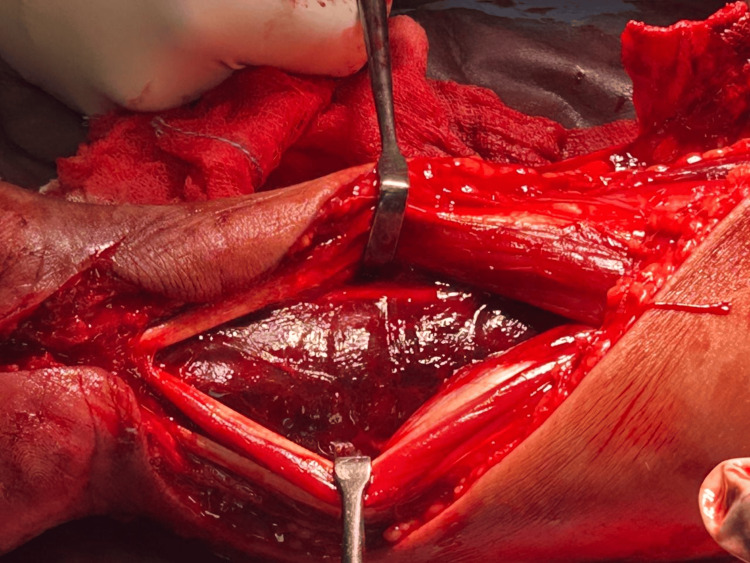
Intraoperative photograph highlighting the dusty appearance of the flexor digitorum profundus muscle

**Figure 2:**
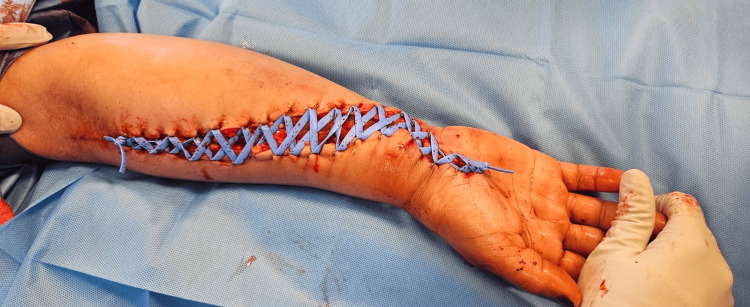
Clinical photograph of the forearm after surgery showing the fasciotomy wound and the "shoelace" type closure technique

An urgent volar forearm fasciotomy was performed. Upon decompression, the flexor digitorum superficialis muscle was mildly dusky but responsive to stimulation. Critically, the flexor digitorum profundus muscle was found to be non-contractile and dusky (Figure 1), consistent with significant ischemic compromise. The flexor pollicis longus and pronator quadratus muscles, however, appeared normal. A carpal tunnel release was also performed, followed by copious irrigation of the compartment. Hemostasis was achieved, and the wound was initially managed with the shoelace closure technique (Figure 2), which accommodates further swelling and facilitates subsequent re-examinations. Definitive wound closure was achieved on postoperative day six. Distal perfusion was carefully assessed and restored post-procedure, with palpable pulses maintained.

Postoperative course

The patient's postoperative course involved three further re-explorations under anesthesia for wound debridement and assessment of muscle viability. She experienced significant intraoperative blood loss during the procedures, necessitating a blood transfusion, but remained hemodynamically stable throughout her admission. After a total hospital stay of 10 days, she was discharged in a stable condition with a prescription for analgesics and a course of antibiotics.

Follow-up

At a two-week outpatient follow-up appointment, the patient demonstrated good functional recovery with improved range of motion and strength. She was formally referred to occupational therapy for a structured physiotherapy regimen to maximize the recovery of strength and mobility in her right hand and forearm.

## Discussion

This case represents an exceptionally rare and intriguing presentation of spontaneous anterior forearm compartment syndrome in a healthy postpartum woman. Its uniqueness stems from the complete absence of traditional triggers such as trauma, vascular injury, or systemic disorders.

The pathophysiology of spontaneous compartment syndrome remains incompletely understood. In this patient, several postpartum-specific physiological and mechanical factors could have contributed. The hypercoagulable state of the postpartum period, which can persist for up to 12 weeks, is a potential contributor that may predispose to microvascular thrombosis within the compartment, increasing intra-compartmental pressure [5]. Furthermore, hormonal changes, particularly relaxin's effects on connective tissue, as well as possible fluid shifts in the postpartum period, may alter compartment compliance and susceptibility. It is plausible that a minor, unremembered compression of the forearm during sleep, which would be inconsequential in a non-puerperal state, acted as the final insult in this susceptible environment.

Our case contrasts with and expands upon the existing literature. Non-traumatic compartment syndromes are most frequently reported in the context of metabolic myopathies like McArdle disease, where exercise-induced rhabdomyolysis is the primary mechanism [2]. While atraumatic forearm cases have been reported, they often occur in athletes or individuals after strenuous activity [3]. Postpartum ACS is itself rare and, as noted in a case report by Coulton et al., typically affects the lower limbs and is often associated with prolonged labor or cesarean section positioning [4]. The occurrence in the upper limb, specifically the forearm, in a patient two months after an uncomplicated delivery, makes this presentation highly unusual.

The diagnosis of ACS in this context was particularly challenging due to the lack of a clear inciting event. This underscores the principle that ACS is primarily a clinical diagnosis. Key signs such as pain disproportionate to the clinical findings and tense swelling in a compartmental distribution must be heeded, even when distal pulses remain intact, as these pulses are often preserved until the very late stages of the syndrome [1]. In such ambiguous cases, direct measurement of intra-compartmental pressure can be a valuable adjunct to clinical judgment. The definitive management, as in all cases of ACS, remains timely and adequate surgical fasciotomy, which was crucial in preserving limb function in our patient.

## Conclusions

This report illustrates that spontaneous anterior compartment syndrome can occur in postpartum patients even in the absence of trauma or identifiable systemic risk factors. Clinicians should be aware of the postpartum period as a potential, albeit rare, risk state for this condition. A high index of suspicion, prompt recognition based on clinical signs, and urgent surgical fasciotomy are vital to prevent permanent functional loss and catastrophic outcomes. This case adds to the limited body of literature on atraumatic ACS and emphasizes the importance of clinical vigilance in atypical contexts.
